# Brazil nuts intake improves lipid profile, oxidative stress and microvascular function in obese adolescents: a randomized controlled trial

**DOI:** 10.1186/1743-7075-8-32

**Published:** 2011-05-28

**Authors:** Priscila A Maranhão, Luiz G Kraemer-Aguiar, Cecilia L de Oliveira, Maria CC Kuschnir, Yasmine R Vieira, Maria GC Souza, Josely C Koury, Eliete Bouskela

**Affiliations:** 1 Clinical and Experimental Research Laboratory in Vascular Biology - BioVasc; Rua São Francisco Xavier, 524, Rio de Janeiro, CEP:20550-013 - Brazil; 2 Endocrinology, Department of Internal Medicine; Clinical and Experimental Research Laboratory in Vascular Biology - BioVasc; Rua São Francisco Xavier, 524, Rio de Janeiro, CEP:20550-013 - Brazil; 3 Nutrition Applied Department; Nutrition Institute - Rua São Francisco Xavier, 524, Rio de Janeiro, CEP: 20550-013 - Brazil; 4 Study Center for Adolescent Health - NESA Av 28 de setembro,. 87, CEP: 20551-030, Rio de Janeiro - Brazil; 5 Physiological Sciences Department; Clinical and Experimental Research Laboratory in Vascular Biology - BioVasc; Rua São Francisco Xavier, 524 , Rio de Janeiro CEP:20550-013 - Brazil; 6 Study Center for Nutrition and Oxidative Stress; Nutrition Institute; Rua São Francisco Xavier, 524, Rio de Janeiro, CEP: 20550-013 - Brazil; 7 Physiological Sciences and Clinical Medicine Departments; Clinical and Experimental Research Laboratory in Vascular Biology - BioVasc; Rua São Francisco Xavier, 524 Rio de Janeiro, CEP:20550-013 -Brazil

**Keywords:** microcirculation, obesity, Brazil nuts, adolescents, oxidative stress, lipid profile

## Abstract

**Background:**

Obesity is a chronic disease associated to an inflammatory process resulting in oxidative stress that leads to morpho-functional microvascular damage that could be improved by some dietary interventions. In this study, the intake of Brazil nuts (*Bertholletia excelsa*), composed of bioactive substances like selenium, α- e γ- tocopherol, folate and polyunsaturated fatty acids, have been investigated on antioxidant capacity, lipid and metabolic profiles and nutritive skin microcirculation in obese adolescents.

**Methods:**

Obese female adolescents (n = 17), 15.4 ± 2.0 years and BMI of 35.6 ± 3.3 kg/m^2^, were randomized 1:1 in two groups with the diet supplemented either with Brazil nuts [BNG, n = 08, 15-25 g/day (equivalent to 3 to 5 units/day)] or placebo [PG (lactose), n = 09, one capsule/day] and followed for 16 weeks. Anthropometry, metabolic-lipid profiles, oxidative stress and morphological (capillary diameters) and functional [functional capillary density, red blood cell velocity (RBCV) at baseline and peak (RBCV_max_) and time (TRBCV_max_) to reach it during post-occlusive reactive hyperemia, after 1 min arterial occlusion] microvascular variables were assessed by nailfold videocapillaroscopy at baseline (T0) and after intervention (T1).

**Results:**

T0 characteristics were similar between groups. At T1, BNG (intra-group variation) had increased selenium levels (*p *= 0.02), RBCV (*p *= 0.03) and RBCV_max _(*p *= 0.03) and reduced total (TC) (*p *= 0.02) and LDL-cholesterol (*p *= 0.02). Compared to PG, Brazil nuts intake reduced TC (*p *= 0.003), triglycerides (*p *= 0.05) and LDL-ox (*p *= 0.02) and increased RBCV (*p *= 0.03).

**Conclusion:**

Brazil nuts intake improved the lipid profile and microvascular function in obese adolescents, possibly due to its high level of unsaturated fatty acids and bioactive substances.

**Trial Registration:**

**Clinical Trials.gov **NCT00937599

## Background

Worldwide prevalence of obesity in adolescence is actually high and increasing [1]. In Brazil, about 29% of adolescents tested between 2008-2009 have excessive weight [2]. Obesity, especially abdominal, even in young subjects leads to metabolic alterations, such as insulin resistance and dyslipidaemia, increasing risk factors for cardiovascular disease (CVD) in adulthood [3].

Excessive abdominal adiposity is characterized by accumulation of adipose tissue and is associated to a low-grade inflammatory process and oxidative stress, both well-established pathogenetic factors for cardiovascular diseases [4]. Morpho-functional microvascular alterations related to metabolic disorders have already been described and findings on skin were related to microvascular dysfunction on coronary bed [5], occurring even in the absence of disglycaemic states [6] pointing to obesity *per se *as independent risk factor for microangiopathy. Possibly, longer duration of excessive adiposity is involved on it as well. Recently, we have unraveled that overweight/obese young women have microvascular dysfunction linked to adiposity levels and glucose homeostasis [7]. Certainly, obesity in adolescence and its long-term damage to target organs in adulthood deserves special focus and strategies to reduce future cardiovascular risks.

Excessive visceral fat induces the release of cytokines, such as tumor necrosis factor-alfa (TNFα) and interleukin-6 (IL-6) leading to increased production of reactive oxygen species (ROS) and subsequent induction of tissue oxidative stress [8]. Some authors consider this pathophysiological process as a major mechanism underlying cardiovascular obesity-related comorbidities [9]. The antioxidant system is directly linked to environmental factors and nutrient intake. Some minerals such as selenium are also involved in decreasing levels of hydrogen peroxide and reducing lesions to cellular membranes [10]. Altered metabolic pathway of very low density lipoprotein cholesterol (VLDL) secondary to excessive visceral adiposity results on higher levels of low density lipoprotein (LDL) particles, which are more aggressive to endothelium. Additionally, the oxidative stress increases oxidation of these LDL particles[11], a process identified as a risk factor for atherosclerosis [12].

Bioactive substances existing in nuts have already been identified [13] and their beneficial effects on inflammation [14] and on endothelial function [15] demonstrated. The Brazil nut (*Bertholletia excelsa*) comes from the Amazon region and has a complex matrix, composed of bioactive substances, such as selenium, α- e γ- tocopherol, phenolic compounds, folate, magnesium, potassium, calcium, proteins and mono (MUFA) and polyunsaturated (PUFA) fatty acids [14]. Its composition is different from other nuts and data to corroborate its beneficial effects, especially in obese adolescents with special focus on microcirculatory function are lacking.

The aims of this study were to investigate the influence of Brazil nuts consumption on nutritive skin microcirculation, serum antioxidant capacity, lipid and metabolic/cardiovascular risk profiles in obese female adolescents.

## Subjects and Methods

### Study Population

Seventeen female adolescents (15.4 ± 2.0 years) were selected, after spontaneous interest to participate (male adolescents showed very little interest), from outpatient care clinics for Prevention and Assistance on Cardiovascular and Metabolic Disease in Adolescence (NESA, State University of Rio de Janeiro, Brazil). Main inclusion criterion was being above 95^th ^percentile for BMI according to age [16]. Main exclusion criteria were: use of any nutritional intervention and/or drugs; presence of chronic diseases (diabetes mellitus and/or hypertension) or lactose intolerance; a verbally informed weight reduction six months before entering the study; being beyond stage IV for Tanner pubertal development [17] and dietary habits of an excessive consumption of any kind of nut. All volunteers gave their written informed consent and this study was approved by the Ethics Committee for Clinical Research of Pedro Ernesto University Hospital (COEP 1950/2007).

### Experimental Design

The study was a 16-week non-blinded pilot trial with two randomly selected groups of obese female adolescents: Brazil nut (BNG, n = 08) ingested 15-25 g/day (equivalent to 3 to 5 units/day) of Brazil nuts and placebo (PG, n = 09) one capsule/day containing lactose. Lactose was chosen as placebo due to its lack of therapeutic effects to improve adolescents' compliance to the study. Both groups were informed that they would receive a supplemental dietary intake composed of Brazil nuts but only one group would receive it on its natural form. Nuts were consumed as snacks or with meals in salads. Before the beginning and at the end of the study, the usual food intake of each participant was assessed by a dietary inquiry and, during the study, adolescents were advised not to change their dietary habits.

Anthropometry, blood, urine and microvascular parameters were analyzed at baseline (T0) and after 16 weeks (T1).

### Brazil Nuts Diet

Brazil nuts (*Bertholletia excelsa*) consumption was calculated to achieve 10% of the energy from MUFAs in the diet. Total energy intake was calculated by energy expenditure to overweight children and adolescents of 3 to 18 years according to dietary reference intake (DRI). Adolescents received 15-25 g/day (equivalent to 3 to 5 units/day) in bags. Serum selenium levels and returned empty bags were used as markers of compliance for BNG.

Brazil nut composition was determined in 100 g by Adolph Lutz Institute-Brazil assays (1985). Lipid, carbohydrates and protein content (mean ± SD) was 50.6 ± 0.08 g [coefficient of variation (CV) 1.6%], 25.9 ± 0.6 g (CV 2.4%) and 16.8 ± 0.2 g (CV 1.4%). Selenium was measured by flame atomic absorption spectrometry. Saturated (SFA - 15.3 g), monounsaturated (MUFA - 27.4 g) and polyunsaturated (PUFA) fatty acids (21 g) contents were calculated according to Brazilian Table of Food Composition [18]. Therefore, nut intake (15-25 g/day) was about: 124 ± 31 kcal with 5.2 ± 1.3 g of carbohydrates and 10.1 ± 2.5 g of lipids. The latter was composed of 4.2 ± 1.0 g of PUFA, 5.5 ± 1.4 g of MUFA and 3.0 ± 0.7 g of SFA and 108.5 ± 27 μg of selenium.

### Anthropometry

The same trained examiner collected anthropometric measurements in duplicate, waist circumference, height, weight as previously reported [19]. BMI was defined as the ratio between weight in kg and squared height in meters.

### Laboratory Analysis

All laboratory measurements were performed in duplicate after 10-12 hours fast using an automated method (Modular Analytics E 170 and P, Roche, Basel, Switzerland). Fasting plasma glucose (FPG), total cholesterol (TC), triglycerides (TG) and high-density lipoprotein (HDL) cholesterol were measured respectively, by enzyme-colorimetric GOD-PAP [inter-assay coefficient of variation (IACV) = 1.09%], enzymatic GPO-PAP (IACV = 2.93%), enzymatic GPO-PAP (IACV = 1.29%) and enzyme-colorimetric without pre-treatment (IACV = 3.23%). Plasma LDL-cholesterol was calculated according to Friedwald equation [20]. C-reactive protein and serum insulin were respectively measured by imunoturbidimetry (IACV = 8%) and eletrochemiluminescence (IACV = 10.6%). Homeostasis model assessment (HOMA-IR) was calculated (fasting serum insulin (μUI/ml) X FPG (mmol/l)/22.5). Serum antioxidant capacity was determined by glutathione peroxidase (GPx) through Elisa [GPX3 (human) Elisa Kit, Axxora, LLC, USA, diluted in 1:200 (IACV = 3.9%; sensitivity = 0.1 ng/ml]. Oxidized-LDL (ox-LDL) levels were also analyzed through Elisa (Kit Mercodia, Sweden), diluted in 1:6561 (IACV = 6.13%; sensitivity = 0.05 ng/ml). Oxidative stress was measured by a competitive enzyme-linked immunosorbant assay in duplicate determining levels of isoprostane nominated as 8-epi-prostaglandin F_2α _(8-epi-PGF_2α_) in urine samples (BIOXYTECH urinary 8-epi-PGF_2α _- OxisResearch, Portland, OR, USA). Urinary samples were acidified during collection with HCl 6 mol/l (final pH = 2.0), diluted to 1:2 before measurements and results were corrected for creatinine levels in each sample assessed by enzymatic colorimetric Modular Evo (Roche) with an intra-assay CV of 5% (2-14%) [19] Serum selenium was determined through atomic absorption spectrometry using a SpectraAA - 640Z - VARIAN (IACV = 11%).

### Microvascular Function Assessment

Nailfold videocapillaroscopy (NVC) was carried out and analyzed according to a standardized, well-validated methodology on the 4^th ^finger of the left hand [7]. The exam, always made by the same observer who was not aware of any patient data, recorded continuously microvascular parameters for later measurements using the Cap Image software [21]. Functional capillary density (FCD), number of capillaries/mm^2 ^with flowing red blood cells, was evaluated using x250 magnification and an area of 3 mm of the distal row of capillaries into three different areas [Intra-assay coefficient of variation - IACV = 5.5 ± 2.5%]. Capillary diameters [afferent (AF), apical (AP) and efferent (EF)], red blood cell velocity (RBCV) at rest, after 1 min arterial occlusion (RBCV_max_) and time taken to reach it (TRBCV_max_) were measured, with a final magnification of x680, before and during the post-occlusive reactive hyperemia (PORH) response after 1 min ischemia. Before RBCV assessment on an individual capillary loop, a pressure cuff (1 cm wide) was placed around the proximal phalanx and connected to a mercury manometer. Conceptually, AF, AP and EF are considered morphological and FCD, RBCV, RBCV_max _and TRBCV_max _functional microcirculatory parameters. Capillary diameters and basal RBCV were measured three times each and IACV for all measurements ranged from 16.9 to 17.1%. At PORH, each variable was tested once. NVC was repeated on nine subjects in different days and the IACV ranged from 12.3% to 17.3% and from 2.0% to 9.0% between morphological and functional parameters, respectively.

### Statisticals Analysis

Data are expressed as median [1^st^-3^rd^] and analyzed by Graphpad Prism 4.0, 2003 and intra- or inter-group analysis compares significant results in different time points within the same group or between BNG and PG, respectively. Comparisons between groups at T0 and T1 and intra-group differences were determined using Mann-Whitney U test and Wilcoxon matched pair tests, respectively. GPower 3.1.10 software was used for power analysis and sample size estimation. The statistical power for comparisons between two dependent groups was above 0.9 with an a error probability of 0.01 for RBCV, estimating a total sample size of 7 patients/group. Significant differences were assumed to be present at *p *< 0.05.

## Results

Adolescents included in the study had 15.4 ± 2.0 years and BMI of 35.6 ± 3.3 kg/m^2^. At T0, there were no significant differences between groups (inter-group) on anthropometrical-laboratorial-microvascular variables (tables 1, 2 and 3). On the counterpart, at T1, we observed lower values for total (*p *= 0.003) and LDL-cholesterol (*p *= 0.03), and also for TG (*p *= 0.05) for BNG compared to PG.

**Table 1:** Anthropometric measurements and metabolic profile of obese female adolescents at baseline and after 16 weeks of Brazil nuts (BNG) or placebo (PG) intake

	BNG		PG	
	**T0**	**T1**	**T0**	**T1**

**Body mass (kg)**	86.3 [82.2-94.3]	86.3[80.3-96.9]	91.7 [82.1-110.8]	93.2[83.7-108.7]
**Height (m)**	1.55[1.52-1.66]	1.55[1.53-1.67]	1.64[1.56-1.69]	1.64[1.56-1.69]
**BMI (kg/m**^**2**^**)**	35.3[33.9-36.0]	35.3[33.3-36.2]	34.0[33.0-39.1]	35.6[32.8-38.9]
**Waist circumference (cm)**	105.0[93.0-117.8]	112.0[99.5-116.0]	111.0[104.0-117.8]	115.0[107.5-120.0]
**Insulin (mcU/ml)**	15.9[14.5-23.6]	15.5[12.2-22.2]	18.0[15.2-20.1]	18.0[10.9-22.8]
**Fasting glucose (mg/dl)**	89.5[81.5-94.0]	86.0 [82.5-93.0]	87.0[82.0-90.5]	90.0[82.0-94.0]
**HOMA**	3.6[2.9-5.2]	3.5[2.6-6.5]	3.9[3.1-4.4]	3.7[2.2-5.2]
**CRP (mg/dl)**	0.31[0.15-0.63]	0.37[0.20-0.68]	0.38[0.14-0.78]	0.52[0.29-0.79]
**Cholesterol (mg/dl)**	152.0[140.5-159.0]	136.0[129.5-141.5] *†	167.0[133.5-184.5]	170.0[148.5-177.0]
**HDL-c (mg/dl)**	44.0[41.5-48.5]	43.5[40.0-50.5]	45.0[36.0-48.0]	45.0[41.0-47.5]
**LDL-c (mg/dl)**	86.5[80.5-98.0]	72.5[71.0-83.0] *†	106.0[77.0-123.0]	103.0[87.0-109.0]
**TG (mg/dl)**	84.5[68.0-98.5]	69.0[58.5-83.5]†	113.5[87.0-132.5]	106.0[77.0-153.0]

* significant results intra-group (p < 0.05)

† significant results inter-group (p < 0.05)

**Table 2:** Biomarkers with antioxidant capacity in obese female adolescents at baseline and after 16 weeks of Brazil nuts (BNG) or placebo (PG) intake

	BNG		PG	
	**T0**	**T1**	**T0**	**T1**

**8-epi-PGF**_**2α **_**(pg/μmol/g of creatinine)**	143.2[102.9-198.8]	100.0[80.0-131.4]	34.0 [61.5-126.6]	88.4[65.0-149.5]
**LDL-ox (ng/ml)**	622.4[457.2-665.0]	514.9[440.3-624.6]†	648.8[515.9-737.9]	646.9[595.7-883.5]
**GPX-3 (ng/ml)**	15.5[12.4-19.8]	16.7[12.8-17.3]	17.2[13.7-19.7]	17.3[13.9-20.1]
**Selenium (μg/L)**	110.5[87.5-131.5]	133.0[104.5-178.0] *	118.0[107.5-148.5]	126.0[106.5-146.5]

* Signicant intra-group results (*p *< 0.05)

† Signficant inter-group results (*p *< 0.05)

**Table 3:** Microcirculatory parameters on obese female adolescents at baseline and after 16 weeks of Brazil nuts (BNG) or placebo (PG) intake

	BNG		PG	
	**T0**	**T1**	**T0**	**T1**

**Functional capillary density (n/mm**^**2**^**)**	10.2[7.8-11.2]	8.8[8.2-11.8]	9.9[8.0-17.4]	10.2[8.4-13.4]
**Afferent diameter (μm)**	15.7[13.9-18.6]	15.2[11.8-19.5]	15.1[12.9-18.6]	14.0[11.6-16.1]
**Apical diameter (μm)**	19.7[18.1-22.3]	22.1[18.2-24.1]	21.9[18.5-27.5]	20.0[18.4-25.6]
**Efferent diameter (μm)**	20.4[17.5-22.9]	21.6[19.2-25.3]	18.4[15.9-21.5]	21.5[17.8-21.8]
**RBCV (mm/s)**	1.43[1.38-1.48]	1.63[1.6-1.7] *†	1.47[1.41-1.52]	1.54[1.40-1.56]
**RBCV**_**max **_**(mm/s)**	1.68[1.64-1.73]	1.84[1.81-1.94] *	1.71[1.56-1.79]	1.79[1.76-1.84]
**TRBCV**_**max **_**(s)**	7.5[6.0-8.0]	4.0[4.0-7.0]	7.5[5.5-8.5]	5.5[5.0-7.0]

* Significant results intra-group (*p *< 0.05)

† Significant results inter-group (*P *< 0.05)

Body mass, BMI, waist circumference and metabolic profile were all kept unchanged during the follow-up on both groups. Additionally, we have noticed that Brazil nuts consumption did not influence glucose homeostasis (FPG, insulin, HOMA-IR) or CRP levels, although its positive influence was observed on LDL- (*p *= 0.01) and total cholesterol (*p *= 0.01) levels (table 1).

**Figure 1:**
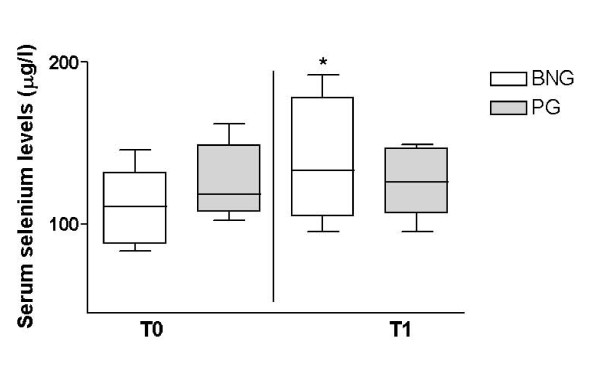
**Differences in serum selenium levels observed on female obese adolescents during follow-up in both groups**. *p = 0.02 (intra-group) BNG = Brazil Nuts Group; PG = Placebo Group.

At T0, antioxidant capacity biomarkers were similar between groups. Gpx, LDL-ox and 8-epi-PGF_2α _did not change during the follow-up in either group (Table 2). It should be highlighted that at T1, serum levels of LDL-ox were lower on BNG when compared to PG (*p *= 0.02), while on PG, serum selenium levels, a marker of compliance for the proposed intervention, was kept unchanged [118 (107.5-148.5 μg/l) vs. 126 (106.5-146.5 μg/l), *p *= 0.74] but on BNG we have observed a significant increase [110.5 (87.5-131.5 μg/l) vs. 133 (104.5-178 μg/l), *p *= 0.02] (Figure 1).

**Figure 2:**
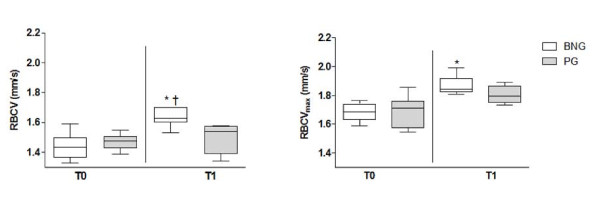
**Resting Red Blood Cell Velocity (RBCV) and RBCV**_**max **_**during post-occlusive reactive hyperemia response (PORH) on obese female adolescents at baseline and after 16 weeks of Brazil nuts (BNG) or placebo (PG) intake**. *p = 0.03 (intra-group) and †p = 0.03 (inter-group).

Morphological microvascular parameters (capillary diameters - Table 3) did not change during the follow-up in both groups. Obese female adolescent in PG had all functional microvascular parameters unaltered during the follow-up but those supplemented with Brazil nuts (BNG) increased RBCV and RBCV_max _during PORH 13.9% and 9.52%, respectively (intra-group results) (Figure 2). Additionally, RBCV at T1 was higher on BNG when compared to PG at the same time point [1.63(1.6-1.7) vs. 1.54 (1.4-1.56) mm/s; *p *= 0.02].

## Discussion

Obesity is associated to metabolic disturbances, including insulin resistance, dyslipidemia and low-grade inflammation, all of them putative factors for endothelial and microvascular dysfunction (MD). Frequent nut intake is associated with many health benefits in adults [22-24], although no studies on adolescents could be found. To the best of our knowledge, this study is the first one to investigate effects of Brazil nuts intake on metabolic-lipid profiles, antioxidant status and microvascular parameters in obese female of this specific age period.

Nuts are rich in lipids, mainly in MUFAs and PUFAs and have high energy density. Comparing fat composition of diets rich on SFAs, high content of MUFAs and PUFAs in foodstuffs is potentially beneficial to health [25]. In spite of the caloric composition, some authors have demonstrated that consumption of nuts for short periods (less than 4 weeks) did not increase body mass[26]. In the present study, both groups kept body mass, waist circumference and BMI unchanged suggesting that although Brazil nuts have high energy density, possibly the satiety feeling due to nuts composition, medium-chain-triglycerides[27], fiber and protein [26] reduced energy intake from other sources. Positive influences on lipid profile by other types of nuts have been already demonstrated [28]. It has been reported that consumption of Brazil nuts during 15 days did not improve LDL- and total cholesterol and its benefits were noticed on transfer of cholesterol into HDL pool. In our study, supplementation of Brazil nuts during 16 weeks to obese female adolescents positively influenced the lipid profile such as, total cholesterol (TC), LDL-C and TG, but the present study did not assess cholesteryl esters.

MD has already been described in obesity [29], metabolic syndrome at normoglycemia [19] and type 2 diabetes mellitus using NVC. This technique is a non-invasive method to assess microvascular morphology and function and data obtained using this diagnostic tool has been already associated to cardiovascular risk [30]. In our investigation, adolescents were seen every 4 weeks what is roughly during the same phase of the menstrual cycle and no especial precaution were taken in this direction because it has already been shown that microvascular function is not dependent on menstrual cycle in ovulatory women [31]. Even tested for short-term period in obese female adolescents, we could observe an improvement of microvascular reactivity by supplementing their diet with Brazil nuts and to our knowledge this is the first clinical study that showed its positive influence on nutritive skin microvascular function, although the effects of PUFAs and MUFAs contained in nuts have been well established on macro and microvascular functions [15].

Capillary morphology was not influenced by Brazil nuts consumption. The analysis of the hemodynamic behavior of the microcirculation was pursued by measuring RBCV before and after 1 min arterial occlusion as well as the time taken to reach RBCV_max_. We have chosen 1 min arterial occlusion because our purpose was to measure the effect of an increased shear stress during the reactive hyperemia response and to decrease the discomfort for our patients (1 min ischemia is more tolerable than 4-6 min) [32]. Longer duration of the occlusion is commonly used to evaluate capillary recruitment [33]. In the BNG, a significant improvement on RBCV at baseline and during PORH could be detected in conjunction with a trend towards a positive influence on time for reperfusion (TRBCV_max_), which needs further elucidation. PORH is thought to be determined at the level of small arterioles [34] and to be independent of the autonomic nervous system [35]. After the onset of reperfusion there is a sharp rise in blood flow followed by a gradual return to its baseline level, influenced by accumulation of vasodilator metabolites (including nitric oxide) and formation of ROS, normally washed out or destroyed by circulating blood and smooth muscle cell reactivity. During reperfusion, the myogenic response, due to rapid stretch of microvascular smooth muscle cells, is responsible, at least partially, for the return of blood flow to its baseline values. Our sample size did not allow us to correlate variables to find out associations between them, but we suppose that higher intake of MUFAs and PUFAs by obese adolescents on BNG resulting in an improved lipid profile and lower levels of LDL-ox, positively influenced microvascular reactivity. To strengthen this hypothesis we have previously noticed a multiple association between total and LDL-cholesterol levels and capillary microflow on obese metabolic syndrome subjects [19], suggesting that interventions with beneficial effects on the lipid profile could positively influence RBCV and RBCV_max_. Consumption of nutrients rich in PUFAs (especially linolenic acids) such as fish oil and also Brazil nuts was associated to reduction in fibrinogen levels and in blood viscosity[36].

Nuts as sources of MUFAs have also been inserted in diets aiming to improve not only lipid profile but also insulin sensitivity, but in healthy, type 2 diabetes mellitus patients [37], and our own results in obese adolescents, nuts-enriched diets did not influence glucose homeostasis and CRP, an acute-phase protein, associated to atherosclerosis, metabolic disorders and CV disease [38].

Brazil nuts consumption during 15 days increased selenium levels, showing that shorter periods with higher amounts of nuts per day were followed by increments of 370% on selenium levels [24]. Physiological values for selenium ranged from 53 to 161.1 μg/l [39]. In the present study, selenium levels increased on BNG and were considered as a marker of good compliance although a significant difference could not be found inter-group (perhaps the intervention time was too short). Oxidative imbalance has been pointed as a causal factor for CVD. Nutrients and bioactive substances with antioxidant action are able to avoid arachidonic acid oxidation [33] and improvement of lipid peroxidation could reduce plasmatic and urinary levels of 8-epi-PGF_2α_, an oxidative product of arachidonic acid related to cell membranes. Urinary 8-epi-PGF_2α _levels, also called isoprostanes, have shown large variation already at baseline inter- (two adolescents at PG had reduced urinary 8-epi-PGF_2α _levels) and also intra-group in a reduced sample size and no significant changes could be detected on either group. However, we have noticed that its levels in BNG showed a decremental direction while in PG results went in the opposite way. It should be recalled that isoprostanes, derived from the arachidonic acid, are only one form of metabolite related to oxidative stress thus not able to reflect the whole oxidative status on cell membranes. Although our data do not explain improvements in the nutritive microvascular reactivity by an amelioration of urinary isoprostanes, we suggest that this pathophysiological focus deserves further elucidation with the use of other biomarkers of oxidative stress.

While investigated markers of oxidative stress were not altered by Brazil nuts consumption (Table 2), we could notice that LDL-ox levels were significantly reduced in BNG, probably due to reduction on oxidative stress status. The exact mechanism involved on it was not our aim, but the knowledge that LDL-ox is associated to endothelial dysfunction and atherosclerosis rise possible questions about the beneficial long-term effects of Brazil nuts consumption on obesity.

Limitations of the present study warrant mention. Although a power analysis was done to strengthen our results, the reduced number of patients investigated limits our conclusion to the population studied. Although the present study was randomized, it was not blinded and we have to consider that some changes in diet that we were not aware of, might have happened. Long-term beneficial cardiovascular outcomes could be inferred by these data.

Finally, treatment strategies for obese adolescent should focus on lifestyle interventions, aiming weight reduction, even if some supplements with established beneficial effects are added to diets.

## Conclusion

Our results show that short-term intake of Brazil nuts added to diet of an obese female adolescent group did not change body mass or waist circumference, but, as a nutrient rich in bioactive substances, it positively influenced lipid profile and nutritive microvascular reactivity.

## List of Abbreviations

8-epi-PGF_2α_: (8-epi-prostaglandin F_2α_); AF: (Afferent diameter); AP: (Apical diameter); BMI: (Body mass index); BNG: (Brazil Nut group); BP: (Blood pressure); CRP: (C-reactive protein); CV: (Coefficient of variation); CVD: (Cardiovascular disease); EF: (Efferent diameter); FCD: (Functional capillary density); FPG: (Fasting plasma glucose); Gpx: (Glutathione peroxidase); HDL: (High-density lipoprotein); HOMA-IR: (Homeostasis model assessment for insulin resistance); IACV: (Inter-assay coefficient of variation; IL-6: (interleukin-6); IR: (Insulin resistance); LDL: (Low-density lipoprotein); MUFA: (Monousatured fatty acids); NCHS: (National Center of healthy Statistics); NVC: (Nailfold videocapillaroscopy); PG: (Placebo group); PORH: (Post-occlusive reactive hyperemia); PUFA: (Polyunsatured fatty acids); RBCV: (Red blood cell velocity); RBCV_max_: (Peak red blood cell velocity); ROS: (Reactive oxygen species); SFA: (Saturated fatty acids); TC: (Total Cholesterol); TG: (Triglycerides); TNFα: (Tumor necrosis factor-alfa); TRBCV_max_: (Time taken to reach RBCV_max_); VLDL: (Very low density lipoprotein); ω-3: (Linolenic fatty acids); ω-6: (Linoleic fatty acids).

## Competing interests

The authors declare that they have no competing interests.

## Authors' contributions

PM - Performed microvascular analyses, statistical analyses and draft the manuscript; LGK - Participated to draft the manuscript and performed statistical analyses; CL - Participated in study design; MCK- Performed patients selection; YR - Carried out immunoassays; MG - Carried out immunoassays; JK- Performed statistical analyses and participated in study design; EB - Participated in study design and to draft the manuscript. All authors read and approved the final manuscript.
